# Can Artificial Intelligence Replace the Unique Nursing Role?

**DOI:** 10.7759/cureus.51150

**Published:** 2023-12-27

**Authors:** S K Mohanasundari, M Kalpana, U Madhusudhan, Kasturi Vasanthkumar, Rani B, Rashmi Singh, Neelam Vashishtha, Vikas Bhatia

**Affiliations:** 1 Pediatric Nursing, All India Institute of Medical Sciences, Bibinagar, Bibinagar, IND; 2 Physiology, All India Institute of Medical Sciences, Bibinagar, Bibinagar, IND; 3 Psychiatric Nursing, All India Institute of Medical Sciences, Bibinagar, Bibinagar, IND; 4 Community Health Nursing, All India Institute of Medical Sciences, Bibinagar, Bibinagar, IND; 5 Obstetrics and Gynaecology Nursing, All India Institute of Medical Sciences, Bibinagar, Bibinagar, IND; 6 Medical Surgical Nursing, All India Institute of Medical Sciences, Bibinagar, Bibinagar, IND; 7 Community Medicine and Family Medicine, All India Institute of Medical Sciences, Bibinagar, Bibinagar, IND

**Keywords:** human connections, collaboration, patient advocacy, adaptability to dynamic situations, critical thinking, artificial intelligence

## Abstract

Artificial intelligence (AI) is transforming healthcare, offering potential benefits and challenges. In healthcare, AI enhances efficiency, streamlines processes, and supports decision-making. However, challenges include potential errors and biases in algorithms, data privacy concerns, legal and ethical issues, and resistance to change. In nursing, a delicate balance emerges between AI and human touch. While AI aids in data-driven decision-making and administrative tasks, it lacks the emotional intelligence, empathy, and nuanced understanding crucial to nursing care. Nurses excel in critical thinking, adaptability to dynamic situations, patient advocacy, collaboration, and establishing human connections. AI supports these functions by automating routine tasks and offering decision support tools, yet its rigidity in dynamic situations and lack of human touch pose limitations. This review underscores the necessity of careful AI integration in healthcare, acknowledging its advantages while mitigating drawbacks. In nursing, the symbiosis between AI and human qualities is vital. The role of AI should be to complement, not replace, the unique skills and empathetic aspects of nursing care. Addressing concerns related to bias, transparency, data privacy, and professional training is essential for maximizing the benefits of AI in healthcare while preserving the human touch in patient care. This article explores whether AI can replace unique nursing roles.

## Introduction and background

Artificial intelligence (AI) is the simulation of human intelligence by machines, especially computer systems. In general, AI systems work by ingesting large amounts of labeled training data, analyzing the data for correlations and patterns, and using these patterns to make predictions about future states [1].

AI has the potential to change how we live, work, and play, as it can perform tasks much better than humans [2]. The role of AI in the healthcare system is very important, and, at present, it has been effectively used in the healthcare system in several areas. Challenges faced by the Indian health system, such as a shortage of qualified healthcare professionals and infrastructure, poor affordability of increasing healthcare expenses, lack of awareness, and lack of access to healthcare services, along with the availability of private facilities only in and around tier 1 and tier 2 cities, which leads to substantial distances for basic and advanced health services and non-uniform access to healthcare across the country, increase the need for AI in the healthcare system [3].

AI has made significant strides in revolutionizing the healthcare system and improving patient outcomes [2] by saving time, energy, and money in the health sector. It avoids unnecessary visits to the hospital for minor ailments, reduces the burden on outpatient and inpatient departments at tertiary care hospitals, provides specialist-based care for the rural population, avoids misguidance, and connects peripheral hospitals with tertiary-level hospitals through an e-referral system [4].

AI is also integrated into nursing care, and this integration has the advantages of efficiency and data-driven decision-making [5]. However, as nursing care involves a human touch in terms of empathy, compassion, warmth, and emotion, it becomes a matter of concern whether AI can replace the human touch of nurses. The synergy between AI and human nursing care is crucial, where technology supports the work of nurses without replacing their compassion and empathy. This comprehensive review article explores whether AI can replace the unique nursing role.

## Review

Power of artificial intelligence for enhanced patient care

AI holds significant potential to revolutionize healthcare by enhancing efficiency, improving patient care, and advancing various aspects of medical practice. Some key benefits include remote monitoring through telemedicine, virtual health assistants for patient engagement [6], early disease detection through advanced medical image analysis, personalized treatment plans based on genetic and lifestyle data [7,8], predictive analytics for disease outbreaks and patient admissions, early warning systems to identify deterioration in patient conditions [9], and the use of AI-powered robots in surgery to reduce the risk of human error [10]. AI is also instrumental in drug discovery [11], streamlining administrative tasks [12], and data analysis in clinical trials and research [13]. These applications highlight the transformative impact of AI on healthcare delivery.

Challenges and drawbacks of artificial intelligence in patient care

A significant drawback of AI in healthcare is the potential for errors and biases in algorithms. Data bias, where training data does not represent the entire population, can lead to unequal treatment. Algorithmic bias and no creativity, reflecting biases in training data, poses risks of misdiagnoses and lack of generalization. [14] The “black box” problem, lack of transparency in AI decision-making, raises trust issues [15]. Patient privacy concerns; legal and ethical issues [16,17]; dependency on AI leading to skill decline, which makes humans lazy and leads to unemployment [18]; high costs; and resistance to change are additional challenges, while the most common drawback is lack of emotions [19,20]. Careful design, testing, and regulation are crucial to address these concerns and maximize AI benefits in healthcare.

Nursing in the age of artificial intelligence: unique nursing role and artificial intelligence

Nursing is as much an art as it is a science. Nurses combine their scientific knowledge and nursing skills in their everyday work in various situations [21]. The essence of nursing care extends beyond administering medications or monitoring vital signs. It involves actively listening to patients, understanding their fears and concerns, offering comfort during difficult times, and advocating for the patients’ rights [22]. Nurses provide solace and reassurance, which are deeply human qualities [23], that cannot be automated.

Nursing encompasses a multifaceted approach to patient care that extends beyond clinical expertise, integrating empathy, critical thinking, and advocacy. Nurses, drawing from hands-on experience and a holistic perspective, employ practical knowledge to assess and manage patient conditions. Their ethical decision-making considers individual values and cultural factors, fostering trust through direct patient communication. Nurses navigate complex scenarios with adaptability, creativity, and nuanced problem-solving skills, uniquely tailored to each unpredictable patient situation, qualities currently lacking in AI systems [24].

Furthermore, nurses demonstrate unparalleled adaptability in addressing patients’ emotional needs within dynamic healthcare settings. Their deep understanding of human emotions, coupled with critical thinking, allows for complex decision-making while excelling in clear and empathetic communication with patients and their families. In providing holistic care, nurses consider not only the physical but also the emotional, psychological, and social dimensions of patient health, a comprehensive approach currently beyond AI capabilities [25].

Patient advocacy is intrinsic to nursing practice, where nurses champion patients’ rights and autonomy. Establishing strong personal connections with patients and families, nurses build trust and advocate effectively for their needs through clear, empathetic communication. Tailoring care plans to individual preferences and advocating for personalized treatment further exemplify the unique role nurses play in patient advocacy, surpassing the capabilities of AI [26].

Nurses thrive in collaborative healthcare environments, working seamlessly with diverse healthcare professionals to ensure holistic patient care. Through effective communication and interdisciplinary collaboration, nurses optimize each team member’s expertise, acting as advocates for patients within the healthcare team. Their adaptability and facilitation of shared decision-making foster cohesive teamwork, ensuring patient-centered care that considers individual preferences and needs, an aspect of care that AI cannot replicate [27].

Above all, the hallmark of nursing lies in its ability to establish a profound human connection. Rooted in empathy and compassion, nurses form genuine relationships with patients, actively listening, understanding emotional needs, and providing unwavering support during challenging times. This innate ability to connect on a human level sets nurses apart, addressing not only the physical but also the emotional and psychological aspects of patient well-being, an essential aspect of care beyond the capabilities of AI [28].

AI has the potential to transform various aspects of nursing, enhancing patient care, improving efficiency, and supporting healthcare professionals. AI can support nurses in their tasks, but it cannot replace their role as caregivers and advocates for patients.

The special role that nurses play in critical areas of patient care, as well as how AI supports and varies from their responsibilities, is covered in the paragraphs that follow.

Nursing Versus Artificial Intelligence in Critical Thinking and Judgment/Clinical Decision-Making

Nurses have hands-on clinical experience, allowing them to draw from a wealth of practical knowledge in assessing and managing patient conditions. They take a holistic approach, considering not only the physical but also the emotional and psychological aspects of patient care. Nurses make ethical and moral decisions, taking into account individual patient values, beliefs, and cultural factors. They communicate directly with patients, which helps build trust and rapport, enabling better judgment in care decisions. Nurses often encounter complex and unique patient situations so they develop and adopt creative problem-solving skills. AI systems lack the adaptability and creativity that human nurses have when dealing with unpredictable and unique patient scenarios [24,29].

In critical thinking and clinical decision-making, AI can assist by analyzing patient data to identify trends and potential risk factors and provide decision-support tools that offer evidence-based recommendations for nursing care. AI algorithms can analyze data to detect subtle changes in patient conditions early, potentially allowing for early intervention and prevention of complications. Predictive analytics can help in forecasting patient outcomes and planning appropriate interventions. Technologies can automate routine tasks, allowing nurses to focus more on critical thinking and complex problem-solving. [6-13,30]

At the same time, AI lacks human emotions and empathy, making it less capable of understanding and addressing the emotional aspects of patient care. It can inherit biases from the data it is trained on, potentially leading to unfair or discriminatory recommendations. Moreover, AI may oversimplify complex patient conditions or provide recommendations based solely on statistical patterns, missing the broader context [14-20,31].

Nursing Versus Artificial Intelligence in Adaptability to Dynamic Situations

Nurses have a deep understanding of human emotions and can adapt their care to address patients’ emotional needs during challenging and dynamic situations. Nurses possess critical thinking skills that enable them to make complex and nuanced decisions in rapidly changing healthcare settings. Nurses excel in clear and empathetic communication with patients and their families, which is crucial in times of uncertainty and change. They provide holistic care that considers not only the physical aspects of health but also the emotional, psychological, and social dimensions [24,29].

AI excels in real-time data analysis, allowing for quick assessment of changing situations. This can be particularly valuable in monitoring patient conditions and identifying trends that may require immediate attention [6-13,30]. AI systems operate based on predefined algorithms and data, which can make them inflexible when dealing with unexpected or rapidly evolving patient conditions. It cannot understand and respond to the emotional needs of patients and their families during dynamic situations. AI heavily relies on the quality and quantity of data available. In situations with limited or biased data, it may provide inaccurate recommendations [14-20,31].

Nursing Versus Artificial Intelligence in Patient Advocacy

Nurses advocate for the rights and autonomy of patients. This includes respecting the patient’s right to make decisions about their care, maintaining confidentiality, and ensuring informed consent. Nurses establish strong personal connections with patients and their families, which helps build trust and advocate effectively for their needs. Nurses excel in clear and empathetic communication, ensuring that patients’ voices are heard and understood. They can tailor care plans to individual patient preferences and advocate for personalized treatment [24,29].

AI can provide patients with access to a wealth of information about their health conditions, treatment options, and lifestyle choices. This empowers patients to actively participate in decisions about their care. AI systems can analyze patient data to offer personalized health information and recommendations. This can contribute to individualized care plans that align with the patient’s unique needs and preferences. AI can assist healthcare professionals, including nurses, in making evidence-based decisions. This can enhance the quality of care provided to patients, supporting advocacy for the best possible outcomes [6-13,30].

If AI algorithms are trained on biased datasets, there is a risk of perpetuating existing biases in healthcare. This could lead to disparities in the information and recommendations provided to different patient groups. The use of AI in patient advocacy involves the collection and analysis of sensitive health data. Ensuring the security and privacy of this information is crucial to maintaining patient trust and compliance. There is a risk of healthcare professionals, including nurses, becoming overly reliant on AI tools. This could potentially lead to a diminished focus on the human aspects of patient care, such as active listening and empathetic communication. AI systems may generate complex outputs that are challenging for patients to understand. This could result in patients feeling overwhelmed or confused by the information provided, affecting their ability to actively participate in decision-making [14-20,31].

Nursing Versus Artificial Intelligence in Collaboration and Teamwork

Nurses work collaboratively with other healthcare professionals, such as doctors, therapists, and social workers, to ensure holistic patient care. This interdisciplinary collaboration optimizes the expertise of each team member for comprehensive and well-rounded patient support. Nurses excel in effective communication, facilitating clear and open lines of communication within the healthcare team. This ensures that all team members are informed, aligned with their goals, and able to coordinate care efficiently. Nurses often act as advocates for patients within the healthcare team. They communicate patient needs and preferences, ensuring that the care provided is in the best interest of the patient. Collaboration in nursing involves shared decision-making with patients and their families. Nurses engage in discussions about treatment options, ensuring that patients are informed and active participants in their care plans. Nurses are adaptable team members, capable of adjusting to changing circumstances and contributing flexibly to the evolving needs of the healthcare team and patients [24,29].

AI systems can facilitate the integration and sharing of patient data among healthcare team members. This promotes a more cohesive and coordinated approach to patient care, reducing the risk of information gaps. AI can provide decision-support tools that assist healthcare professionals in making well-informed decisions. This can enhance collaboration by ensuring that team members have access to evidence-based information and recommendations. AI technologies can automate routine tasks, allowing healthcare professionals, including nurses, to focus more on collaborative aspects of care. This can streamline workflows and improve overall team efficiency [6-13,30].

AI lacks the human touch and interpersonal skills essential for effective collaboration. The empathetic communication and emotional intelligence brought by human team members, such as nurses, are critical for building trust and understanding. AI systems may struggle to interpret the complexity of certain patient cases that require a deep understanding of individual circumstances and context. Human intuition and clinical judgment are crucial in navigating these intricacies. Overreliance on AI systems without critical evaluation can lead to trust issues within the healthcare team. Human healthcare professionals must maintain a level of skepticism and be capable of interpreting and validating AI-generated information. The use of AI involves the collection and sharing of sensitive patient data. Ensuring the security and privacy of this data is essential to maintaining trust among healthcare team members and protecting patient confidentiality. Healthcare professionals need to be adequately trained in using AI tools. Lack of familiarity or insufficient training can hinder the seamless integration of AI into collaborative healthcare settings [14-20,31].

Nurses Versus Artificial Intelligence in Human Connection

Nursing is deeply rooted in empathy and compassion. Nurses form genuine connections with patients, understanding their emotional needs and providing comfort and support during challenging times. Nurses excel in active listening, ensuring that patients feel heard and understood. This communication skill fosters a strong human connection and allows nurses to address not only the physical but also the emotional and psychological aspects of patient well-being.

Establishing trust is a crucial aspect of nursing. Patients often share sensitive information with nurses, trusting them to maintain confidentiality and provide personalized care. This trust forms the foundation of a strong human connection. Nurses are trained to be culturally competent, respecting and understanding the diverse backgrounds and beliefs of their patients. This cultural sensitivity enhances the ability to form meaningful connections with individuals from various walks of life. Nursing involves considering the patient as a whole person, addressing not only their medical needs but also their social, spiritual, and psychological well-being. This holistic approach strengthens the human connection between nurses and patients [24,29].

AI can provide patients with access to a vast amount of information about their health conditions, treatment options, and lifestyle choices. This empowers patients to actively engage in their care and make informed decisions. AI systems can analyze patient data to offer personalized health information and recommendations. This personalization can contribute to a more tailored and patient-centric approach to care. AI-powered virtual assistants and monitoring systems can offer continuous support to patients, providing information, reminders, and monitoring health parameters. This can enhance the overall patient experience and engagement [6-13,30].

At the same time, AI lacks emotional intelligence and the ability to truly understand and respond to the emotional needs of patients. The nuanced and empathetic communication that is crucial for building a human connection is challenging for AI to replicate. In some cases, the use of AI in healthcare may lead to concerns about dehumanization, where patients feel they are interacting more with machines than with caring individuals. This can potentially impact the quality of the human connection in healthcare. Overreliance on AI for certain aspects of care may result in reduced human interaction, leading to feelings of isolation among patients. Human connection plays a vital role in emotional well-being, and excessive dependence on technology may undermine this aspect of care. The use of AI involves the collection and analysis of sensitive patient data. Concerns about data privacy and security may impact the trust patients place in healthcare systems, affecting the human connection between healthcare providers and patients. The use of AI tools may inadvertently impact the development of communication skills in healthcare professionals. Over time, there could be a risk of diminished emphasis on essential interpersonal skills if technology becomes the primary mode of communication [14-20,31].

## Conclusions

AI holds immense promise in transforming healthcare, offering unprecedented efficiencies and improvements in patient care. The integration of AI in the healthcare system addresses critical challenges faced by the Indian health system, such as a shortage of qualified professionals and infrastructure, limited affordability, and uneven accessibility. The contributions of AI extend beyond administrative tasks to early disease detection, personalized treatment plans, predictive analytics, and even surgical interventions, showcasing its transformative impact on healthcare delivery.

However, the deployment of AI in healthcare is not without its challenges and drawbacks. Concerns include the potential for errors and biases in algorithms, lack of transparency in decision-making (the “black box” problem), patient privacy issues, legal and ethical considerations, dependency leading to skill decline, high implementation costs, and resistance to change within the healthcare ecosystem. To harness the benefits of AI while mitigating these challenges, careful design, testing, and regulation are imperative.

Within the specific context of nursing, this article highlights the unique and irreplaceable role that human touch plays in patient care. While AI can assist in critical thinking, adaptability to dynamic situations, patient advocacy, and collaborative efforts, it lacks the emotional intelligence and nuanced understanding essential in nursing. The human connection formed through empathy, compassion, and cultural competence is a hallmark of nursing care that AI struggles to replicate.

The synergy between AI and nursing is emphasized, with AI serving as a supportive tool rather than a replacement for the intricate and deeply human aspects of nursing. The human touch in nursing, characterized by genuine connections, trust-building, and holistic care, remains indispensable. As the healthcare landscape continues to evolve with technological advancements, maintaining a balance that preserves the essence of the human touch while leveraging the benefits of AI is crucial for achieving optimal patient outcomes and experiences.
